# Urinary beta-2 microglobulin increases whereas TIMP-2 and IGFBP7 decline after unilateral nephrectomy in healthy kidney donors

**DOI:** 10.1038/s41598-024-62246-1

**Published:** 2024-06-05

**Authors:** Esther N. M. de Rooij, Ellen K. Hoogeveen, Fred P. H. T. M. Romijn, Sandra W. van der Kooy, Kristin V. Veighey, Friedo W. Dekker, Cees van Kooten, Christa M. Cobbaert, Johan W. de Fijter

**Affiliations:** 1 Department of Nephrology, Leiden University Medical Center, Leiden, The Netherlands; 2 Department of Clinical Epidemiology, Leiden University Medical Center, Leiden, The Netherlands; 3 Department of Nephrology, Jeroen Bosch Hospital, Den Bosch, The Netherlands; 4 Department of Clinical Chemistry and Laboratory Medicine, Leiden University Medical Center, Leiden, The Netherlands; 5 Wessex Kidney Centre, Portsmouth Hospitals NHS Trust, Portsmouth, Hampshire UK; 6 Research and Development, University Hospital Southampton NHS Foundation Trust, Southampton, Hampshire UK; 7 Department of Nephrology, Antwerp University Hospital, Edegem, Belgium; 8 Laboratory of Experimental Medicine and Pediatrics (LEMP), University of Antwerp, Wilrijk, Belgium

**Keywords:** Biomarkers, Medical research, Nephrology

## Abstract

Early kidney injury may be detected by urinary markers, such as beta-2 microglobulin (B2M), tissue inhibitor of metalloproteinases-2 (TIMP-2), insulin-like growth factor-binding protein 7 (IGFBP7), kidney injury molecule-1 (KIM-1) and/or neutrophil gelatinase-associated lipocalin (NGAL). Of these biomarkers information on pathophysiology and reference ranges in both healthy and diseased populations are scarce. Differences in urinary levels of B2M, TIMP-2, IGFBP7, KIM-1 and NGAL were compared 24 h before and after nephrectomy in 38 living kidney donors from the REnal Protection Against Ischaemia–Reperfusion in transplantation study. Linear regression was used to assess the relation between baseline biomarker concentration and kidney function 1 year after nephrectomy. Median levels of urinary creatinine and creatinine standardized B2M, TIMP-2, IGFBP7, KIM-1, NGAL, and albumin 24 h before nephrectomy in donors were 9.4 mmol/L, 14 μg/mmol, 16 pmol/mmol, 99 pmol/mmol, 63 ng/mmol, 1390 ng/mmol and 0.7 mg/mmol, with median differences 24 h after nephrectomy of − 0.9, + 1906, − 7.1, − 38.3, − 6.9, + 2378 and + 1.2, respectively. The change of donor eGFR after 12 months per SD increment at baseline of B2M, TIMP-2, IGFBP7, KIM-1 and NGAL was: − 1.1, − 2.3, − 0.7, − 1.6 and − 2.8, respectively. Urinary TIMP-2 and IGFBP7 excretion halved after nephrectomy, similar to urinary creatinine, suggesting these markers predominantly reflect glomerular filtration. B2M and NGAL excretion increased significantly, similar to albumin, indicating decreased proximal tubular reabsorption following nephrectomy. KIM-1 did not change considerably after nephrectomy. Even though none of these biomarkers showed a strong relation with long-term donor eGFR, these results provide valuable insight into the pathophysiology of these urinary biomarkers.

## Introduction

After living kidney transplantation, early identification of acute kidney injury (AKI) in both transplant recipients and living donors is key to long-term preservation of kidney function^1^. Delayed graft function (DGF), or AKI due to ischemia and reperfusion injury requiring dialysis within the first week after kidney transplantation, occurs in 25–55% and 1–8% of recipients after deceased and living donor kidney transplants, respectively^2,3^. AKI is defined as a reduced urine output (e.g. urine output < 0.5 mL/kg/h for 6 h) and an increase in plasma creatinine (e.g. increase ≥ 26.5 µmol/L within 48 h)^4–6^. Elevation of plasma creatinine is the most widely used measure of glomerular filtration rate (GFR), but indicates established loss of kidney function rather than early or ongoing tissue injury. Consequently, mild or early kidney damage generally remains unnoticed due to the renal reserve capacity and/or hyperfiltration by the remaining healthy nephrons^4^. In addition, estimated GFR (eGFR) based on plasma creatinine provides no information on the underlying etiology of kidney function decline. There also is a close linkage between AKI and subsequent chronic kidney disease (CKD). AKI has been associated with an increased risk of CKD, recurrent AKI, hospitalizations, cardiovascular morbidity and mortality^7^. AKI can lead to maladaptive regeneration and repair of renal tissue and/or trigger proinflammatory cascades in the kidney that may lead to progressive fibrosis^4,7^.

Several biomarkers have been identified for early detection of kidney damage, both in the general population and in kidney transplantation recipients. However, views on the pathophysiology and clinical value of these biomarker change as research continues. Recently, attention has focused on kidney injury molecule-1 (KIM-1), neutrophil gelatinase-associated lipocalin (NGAL), urinary tissue inhibitor of metalloproteinases-2 (TIMP-2) and insulin-like growth factor–binding protein 7 (IGFBP7). KIM-1 and NGAL have been found to be involved in kidney recovery and tubular regeneration^8,9^. For example, NGAL elevation can already be measured from 3 h after kidney injury^10^. Recently, TIMP-2 and IGFBP7 were also approved by the U.S. Food and Drug Administration for clinical AKI prediction. TIMP-2 and IGFBP7 have been shown to outperform other urinary markers in AKI diagnosis, including KIM-1 and NGAL^11^. However, little is known about the cellular sources and physiological roles of urinary IGFBP7 and TIMP-2^12^. Finally, of all four biomarkers information on reference ranges in both healthy and diseased populations are scarce.

Nearly 50 years after beta-2 microglobulin (B2M) was first shown to be an indicator of reduced GFR, this non-creatinine renal filtration marker was shown to outperform KIM-1 in predicting a low eGFR 1 year after AKI^13,14^. Whether B2M does indeed surpass the novel kidney injury biomarkers in indicating kidney damage and what pathophysiologic differences and similarities exist remains to be studied.

Therefore, we aimed to assess the pathophysiological properties of urinary B2M, TIMP-2, IGFBP7, KIM-1 and NGAL in a healthy cohort of living kidney donors before and after unilateral nephrectomy, to study the effect of a reduction in kidney mass rather than kidney damage. Then, we investigated these markers after the subsequent implantation in their respective recipients, as a model of established ischemia reperfusion injury. Furthermore, we studied whether peri-operative levels of urinary B2M, TIMP-2, IGFBP7, KIM-1 and NGAL were predictive of eGFR 1 year after nephrectomy in otherwise healthy living kidney donors and their recipients. These results provide valuable insight into the pathophysiology of these urinary biomarkers.

## Methods

### Study population

For the current project, we used a cohort of 38 donor–recipients pairs from the REnal Protection Against Ischaemia–Reperfusion in transplantation (REPAIR) study. Briefly, in the original study, patients took part in a multicenter double-blind randomized clinical trial (ISRCTN registry number 30083294) which investigated whether remote ischaemic preconditioning (RIPC) improved renal function after living-donor kidney transplantation. Full details of the REPAIR study have been described previously^15,16^. In brief, between 4 January 2010 and 29 April 2013, 406 live donor–recipient pairs aged ≥ 18 years were included from thirteen tertiary care hospitals in the UK, the Netherlands, Belgium and France. Patients who used medicines that modulate preconditioning pathways (ATP-sensitive potassium channel opening or blocking drugs, or ciclosporine), had iodine sensitivity (contraindicating iohexol), or required antibody removal (ABO- or HLA-incompatible transplants) were excluded. Each donor–recipient pair was randomized to one of four groups: sham RIPC, early RIPC (immediately before surgery), late RIPC (24 h before surgery) and dual RIPC (early and late RIPC). Both donor and recipient received the same intervention (active RIPC or sham RIPC) at the two time points. Patients were followed up to 5 years after transplantation. The REPAIR study showed a sustained improvement in eGFR after early RIPC, compared with control from 3 months to 5 years after living kidney transplantation (adjusted mean difference: 4.71 mL/min/1.73 m^2^ [95% confidence interval, CI 1.54–7.89]; *P* = 0.004)^16^. The REPAIR study was conducted in accordance with the Declaration of Helsinki and approved by the Joint University College London (UCL)/University College London Hospital (UCLH) Committees on the Ethics of Human Research in June 2009 (reference number 09/H0715/48). Local research ethics committee approvals were gained at all recruiting sites and hospitals involved. Written informed consent was obtained from all patients^15^.

### Urine samples and measurements

For the present study, we used heparinised plasma and spot urine samples from 38 out of the original 55 Dutch REPAIR donor–recipient pairs, for whom all samples were available. Donor plasma and urine samples were collected twice, namely 24 h before and 24 h after nephrectomy. Recipient plasma and urine samples were only collected 24 h after kidney transplantation, resulting in a total amount of 112 (= 3 × 38 minus 2 missing) plasma and 114 (= 3 × 38) urine samples. All blood and urine samples were centrifuged at 400*g* for 10 min and the supernatant was aspirated, partitioned into 2 mL aliquots and stored at − 70 °C to − 80 °C. Samples underwent one freeze–thaw cycle and were stored for a maximum of 10 years prior to chemical analysis.

All plasma and urinary measurements were performed from September 2020 to February 2021. Plasma creatinine, cystatin C, albumin, urea, B2M and urinary creatinine, albumin and B2M were measured using a Cobas c502 analyzer (Roche Diagnostics, Mannheim, DE) according to the manufacturer’s instructions. GFR was estimated based on plasma creatinine (eGFR_cr_), plasma cystatin C (eGFR_cysC_) and combined plasma creatinine–cystatin C (eGFR_cr–cysC_), using the Chronic Kidney Disease Epidemiology Collaboration equations from 2012, taking into account age, sex and race^17^. For long-term eGFR at 3 and 12 months after transplantation only eGFR_cr_ was available since these data were extracted from patient files. Urinary osmolality was measured using Osmo-Station OM-6060, ARKRAY Inc., Kyoto, Japan. Liquicheck (BIO-RAD, Irvine, CA) quantitative urine controls were used for internal QC, mean (SD, %CV) values were 310 mOsmol/kg (2 mOsmol/kg, 0.6%, n = 29) and 551 mOsmol/kg (3 mOsmol/kg, 0.6%, n = 29) for normal and abnormal quality control.

Concentrations of urinary TIMP-2, IGFBP7, KIM-1 and NGAL were measured using sandwich enzyme-linked immunosorbent assays (ELISA) according to manufacturer’s instructions (Cat. Nr. DTM200, R&D systems, Minneapolis, MN for TIMP-2, Cat. Nr. EK0991, Boster Biological Technology, Pleasanton, CA for IGFBP7, Cat. Nr. DY1750, R&D systems, Minneapolis, MN for KIM-1 and Cat. Nr. DY1757, R&D systems, Minneapolis, MN for NGAL). Sample dilutions used were checked to be within the linear range, and the same lot numbers (P238376 for TIMP-2, 6371674817 for IGFBP7) were used throughout the entire study. For TIMP-2 and IGFBP7, two internal low and high-level quality control (QC) samples (QC1 and QC2), consisting of pooled urine, were analysed in triplicate on each sample plate to assess the stability of the assay. For TIMP-2, total analytical imprecision, expressed as coefficient of variation % (CV%), was 3.6% at mean (SD) 172 (6) pmol/L for QC1 (*n* = 15) and 4.2% at 243 (10) pmol/L for QC2 (*n* = 15). For IGFBP7 CV% was 9.4% at 757 (71) pmol/L for QC1 (*n* = 20) and 10.9% at 2053 (224) pmol/L for QC2 (*n* = 20).

### Additional data

Baseline characteristics 24 h before kidney transplantation and data on laboratory measurements during follow-up were provided from the original REPAIR study database. Participant characteristics included age, sex, ethnicity, RIPC randomised group, BMI, systolic blood pressure, primary kidney disease and whether there was a history of dialysis or a previous kidney transplantation.

### Statistical analysis

Baseline characteristics of the living kidney transplant donors and recipients are presented as mean with standard deviation (SD) or median with interquartile range (IQR) for continuous data when appropriate. Categorical variables are expressed as proportions. First, in 38 donors 24 h before and after nephrectomy and 38 donor–recipient pairs 24 h after transplantation, mean (SD) and mean differences (95% CI) of plasma creatinine, cystatin C, eGFR_cr_, eGFR_cys_, eGFR_cr-cys_ and B2M and median concentrations (IQR) and differences (95% CI) of urinary albumin-to-creatinine ratio (ACR) were calculated. Mean differences were calculated using the paired *t* test and median differences using the Wilcoxon signed rank test.

Second, median (IQR) concentrations and median differences (95% CI) of urinary B2M, TIMP-2, IGFBP7, KIM-1 and NGAL standardized for urinary creatinine were calculated for the 38 living donor (24 h before and after nephrectomy) and recipient (24 h after surgery) pairs. Results were also visualized with boxplots. Third, we calculated changes between biomarkers measured in donors and recipients 1 day after transplantation, compared to biomarkers measured in donors 1 day before and after transplantation. Fourth, associations between urinary levels of B2M, TIMP-2, IGFBP7, KIM-1 and NGAL and concurrent eGFR_cr-cys_ were assessed using linear regression analysis, both crude and adjusted for donor age and sex, in the 38 living donor (24 h before and after surgery) and recipient (24 h after surgery) pairs. Additionally, using similar models, the associations between change in urinary levels of B2M, TIMP-2, IGFBP7, KIM-1 and NGAL and change in eGFR_cr-cys_ from 24 h before to 24 h after nephrectomy and eGFR _cr-cys_ at 1 year after nephrectomy were assessed in the 38 living donors. In all regression analyses, B2M, TIMP-2, IGFBP7, KIM-1 and NGAL were divided by their SD or log-transformed by the natural logarithm to normalize their distributions.

In all analyses apart from those investigating long-term eGFR, concentrations of urinary B2M, TIMP-2, IGFBP7, KIM-1 and NGAL were corrected for urinary creatinine and osmolality separately and expressed as a ratio of the biomarker divided by creatinine. All analyses were performed using R version 3.5.1 (R Core Team, Vienna, Austria).

## Results

Baseline characteristics of participant are presented in Table 1. In the 38 donor–recipient pairs mean (SD) age was 54 years (± 12) and 48 years (± 16), 46% and 59% were males, respectively. Mean (SD) donor eGFR_cr_ was 86 (± 17) mL/min/1.73 m^2^.

**Table 1:** Baseline characteristics of 38 living kidney donor–recipient pairs.

	Donors *n* = 38	Recipients *n* = 38
Age, years	54 ± 13	48 ± 16
Male, *n* (%)	18 (47)	22 (58)
Ethnicity, *n* (%)		
White	31 (82)	31 (82)
Black	0 (0)	0 (0)
Asian	0 (0)	0 (0)
Other/unknown	7 (18)	7 (18)
Country, *n* (%)		
Dutch	38 (100)	38 (100)
British	0 (0)	0 (0)
RIPC randomised group, n (%)		
Early	6 (16)	6 (16)
Late	9 (24)	9 (24)
Dual	12 (32)	12 (32)
Control	11 (29)	11 (29)
Primary kidney disease, *n* (%)		
Glomerulonephritis	–	3 (8)
Pyelonephritis	–	0 (0)
Diabetes	–	2 (5)
Polycystic kidney disease	–	6 (16)
Hypertension	–	4 (11)
Other/uncertain	–	23 (61)
Height (cm)	173 ± 11	171 ± 11
Weight (kg)	78 ± 13	73 ± 16
BMI (kg/m^2^)	26 ± 3	25 ± 4
Plasma creatinine, μmol/L	78 ± 18	633 ± 263
Plasma cystatin C, mg/L	0.88 (± 0.13)	–
eGFR_cr_ (mL/min/1.73 m^2^)	88 ± 16	–
eGFR_cysC_, mL/min/1.73 m^2^	91 (± 16)	–
eGFR_cr-cysC_, mL/min/1.73 m^2^	90 (± 15)	–
Plasma urea, g/L	5 ± 1	25 ± 8
Plasma albumin, g/L	47 ± 4	43 ± 5

Data are expressed as the mean ± standard deviation (SD) or number (percentage). Laboratory measurements were conducted 24 h prior to nephrectomy or kidney transplantation.

*BMI* body mass index, *BP* blood pressure, *cr* creatinine, *eGFR* estimated glomerular filtration rate, *RIPC* remote ischaemic preconditioning.

### Biomarker levels before and after unilateral nephrectomy

**Figure 1:**
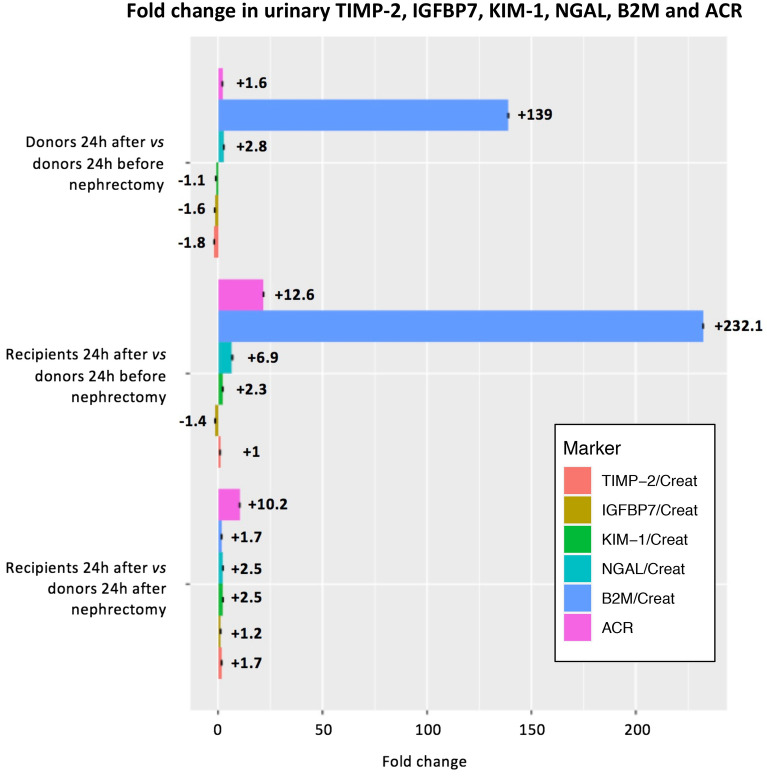
Fold change in urinary creatinine standardized TIMP-2, IGFBP7, KIM-1, NGAL, B2M and albumin measured 24 h after transplantation in 38 living kidney transplantation recipients and donors compared to donors 24 h before and after nephrectomy. *ACR* albumin-to-creatinine ratio, *B2M* beta-2 microglobulin, *IGFBP7* insulinlike growth factor-binding protein 7, *IRI* ischemia–reperfusion injury, *KIM-1* kidney Injury molecule-1, *NGAL* neutrophil gelatinase-associated lipocalin, *TIMP-2* tissue inhibitor of metalloproteinases-2.

Mean (SD) and median (IQR) levels and mean and median differences (95% CI) of urinary creatinine standardized biomarker levels and other relevant laboratory measurements in the 38 donor–recipient pairs are presented in Table 2. Median levels (95% CI) of urinary creatinine standardized B2M, TIMP-2, IGFBP7, KIM-1 and NGAL 24 h before nephrectomy in living donors were 14 (8–21) μg/mmol, 16 (10–22) pmol/mmol, 99 (66–136) pmol/mmol, 63 (33–77) ng/mmol and 1390 (681–2281) ng/mmol, respectively. Median levels (95% CI) of fractional B2M excretion were 0.1 (0.0–0.1)% in donors 24 h before nephrectomy, 10.3 (3.9–13.6) % in donors 24 h after nephrectomy and 23.6 (9.5–39.5) % in recipients 24 h after transplantation. The median difference (95%) in creatinine standardized B2M in donors 24 h after compared to 24 h before renal transplantation was + 1906 (+ 1327 to + 2372) μg/mmol, indicating a 139-fold change. For creatinine standardized TIMP-2, IGFBP7, KIM-1 and NGAL, these median (95%) differences were − 7.1 (− 10.3 to − 4.3) pmol/mmol, –38.3 (− 55.5 to − 24.2) pmol/mmol, − 6.9 (− 21.2 to + 5.1) ng/mmol and + 2378 (− 3621 to + 1146) ng/mmol, indicating fold changes of + 2.8, − 1.1, − 1.6, − 1.8, respectively (Table 2, Fig. 1). Median differences between donors and recipients 24 h after transplantation for creatinine-standardized B2M, TIMP-2, IGFBP7, KIM-1 and NGAL were + 2081 (− 644 to + 3452), + 5.5 (+ 3.3 to + 7.9) pmol/mmol, + 8.8 (− 4.4 to + 23.0) pmol/mmol, + 94 (+ 63 to + 136) ng/mmol and + 4182 (+ 1999 to + 9167) ng/mmol, with fold changes of + 1.7, + 1.7, + 1.2, + 2.5 and + 2.5, respectively (Table 2, Fig. 1). The median difference (95%) in albumin-to-creatinine ratio between donors and recipients 24 h after transplantation was + 13.2 (+ 8.5 to + 18.6) mg/mmol, indicating a 10.2-fold change (Table 2, Fig. 1).

**Table 2:** Kidney function and urinary biomarkers of kidney injury before and after living kidney transplantation in 38 living kidney donor–recipient pairs.

	Donor *n* = 38		Mean or median difference (95% CI)	Recipients *n* = 38	Mean or median difference (95% CI)
			Donors		Donors–recipients
	24 h prior	24 h post	24 h prior–24 h post	24 h post	24 h post
**Plasma**					
Creatinine, μmol/L	76 (± 16)	121 (± 21)	+ 47 (+ 39 to + 50)	219 (± 102)	+ 98 (+ 60 to + 136)
Cystatin C, mg/L	0.88 (± 0.13)	1.24 (± 0.17)	+ 0.36 (+ 0.32 to + 0.40)	1.68 (± 0.64)	+ 0.44 (+ 0.22 to + 0.66)
eGFR_cr_, mL/min/1.73 m^2^	88 (± 16)	52 (± 9)	− 36 (− 40 to − 32)	35 (± 24)	− 16 (− 24 to − 7)
eGFR_cysC_, mL/min/1.73 m^2^	91 (± 16)	60 (± 13)	− 32 (− 36 to − 28)	46 (± 19)	− 13 (− 19 to − 7)
eGFR_cr-cysC_, mL/min/1.73 m^2^	90 (± 15)	55 (± 9)	− 35 (− 39 to − 32)	38 (± 20)	− 17 (− 24 to − 10)
**Urine**					
B2M/Creat, μg/mmol	14 (8 to 21)	1946 (871 to 2637)	+ 1906 (+ 1327 to + 2372)	3250 (1297 to 6769)	+ 2081 (− 644 to + 3452)
TIMP-2/Creat, pmol/mmol	16 (10 to 22)	9 (7 to 11)	− 7.1 (− 10.3 to − 4.3)	16 (11 to 19)	+ 5.5 (+ 3.3 to + 7.9)
IGFBP7/Creat, pmol/mmol	99 (66 to 136)	61 (49 to 81)	− 38.3 (− 55.5 to − 24.2)	73 (50 to 97)	+ 8.8 (− 4.4 to + 23.0)
KIM-1/Creat, ng/mmol	63 (33 to 77)	58 (44 to 86)	− 6.9 (− 21.2 to + 5.1)	144 (108 to 256)	+ 94 (+ 63 to + 136)
NGAL/Creat, ng/mmol	1390 (681 to 2281)	3834 (1870 to 6643)	+ 2378 (− 3621 to + 1146)	9559 (4668 to 24,476)	+ 4182 (+ 1999 to + 9167)
Creatinine, mmol/L	9.4 (3.9 to 12.8)	6.5 (4.3 to 11.0)	− 0.9 (− 3.3 to + 1.3)	5.6 (4.5 to 8.9)	− 0.6 (− 2.4 to + 1.0)
Osmolality, mOsm/kg	557 (318 to 808)	437 (280 to 594)	− 116 (− 237 to − 13)	440 (363 to 503)	+ 2.0 (− 92.0 to + 87.0)
ACR, mg/mmol	0.7 (0.5 to 1.2)	1.5 (1.1 to 2.6)	+ 1.2 (+ 0.5 to + 2.6)	15.3 (8.8 to 32.3)	+ 13.2 (+ 8.5 to + 18.6)
Fractional B2M excretion, %	0.1 (0.0 to 0.1)	10.3 (3.9 to 13.6)	+ 9.0 (+ 6.3 to + 11.5)	23.6 (9.5 to 39.5)	+ 14.1 (+ 6.4 to + 21.0)

Results are presented as mean (± standard deviation), median (interquartile range) or median or mean difference (95% CI).

*ACR* albumin-to-creatinine ratio, *B2M* beta-2 microglobulin, *cr* creatinine, *cysC* cystatin C, *IGFBP7* insulin-like growth factor-binding protein 7, *KIM-1* kidney injury molecule-1, *NGAL* neutrophil gelatinase-associated lipocalin, *TIMP-2* tissue inhibitor of metalloproteinases-2.

*Plasma samples were missing for 1 donor and 1 recipient at 24 h prior to living kidney transplantation.

### Association between biomarkers and short-term eGFR

The regression coefficients (95% CI) for the associations between log-transformed, creatinine standardized urinary levels of B2M, TIMP-2, IGFBP7, KIM-1 and NGAL and concurrent eGFR_cr-cysC_ measurements are presented in Table 3. No clear associations were observed. After adjustment for donor age and sex, TIMP-2 and IGFBP7 showed the highest regression coefficients (Supplemental Table 1).

**Table 3:** Linear regression analysis for association between log-transformed urinary creatinine standardized urinary TIMP-2, IGFBP7, KIM-1 or NGAL, and CKD-EPI eGFR based on plasma creatinine and cystatin C 24 h before and after living kidney transplantation in 38 donor–recipient pairs.

	Crude		
	eGFR_cr-cysC_, mL/min/1.73 m^2^ (95% CI)		
	Donor		Recipient
	24 h prior	24 h post	24 h post
	*n* = 37*	*n* = 38	*n* = 37*
Log(B2M/Creat)	− 8.1 (− 12.4 to − 3.9)	+ 1.0 (− 1.0 to + 3.0)	− 3.3 (− 8.1 to + 1.6)
Log(TIMP-2/Creat)	− 5.0 (− 13.6 to +3.6)	− 2.8 (− 11.3 to + 5.6)	− 0.2 (− 17.0 to + 16.6)
Log(IGFBP7/Creat)	+ 3.3 (− 6.0 to + 12.7)	+ 1.0 (− 6.3 to + 8.3)	+ 6.8 (− 10.2 to +23.8)
Log(KIM-1/Creat)	− 6.3 (− 12.2 to − 0.3)	− 2.5 (− 6.0 to + 1.0)	+ 9.5 (+ 2.0 to + 17.0)
Log(NGAL/Creat)	− 2.1 (− 7.5 to + 3.4)	− 2.8 (− 5.2 to − 0.4)	− 5.8 (− 11.0 to − 0.7)
Log(ACR, mg/mmol)	− 11.4 (− 29.2 to + 6.5)	+ 2.5 (− 2.2 to + 7.1)	+ 4.9 (− 4.3 to + 14.1)

Results are presented as linear regression coefficients (95% CI).

*ACR* albumin-to-creatinine ratio, *B2M* beta-2 microglobulin, *cr* creatinine, *cysC* cystatin C, *IGFBP7* insulin-like growth factor-binding protein 7, *KIM-1* kidney injury molecule-1, *NGAL* neutrophil gelatinase-associated lipocalin, *TIMP-2* tissue inhibitor of metalloproteinases-2.

*Plasma samples were missing for 1 donor at 24 h before and 1 recipient at 24 h after living kidney transplantation.

### Association between biomarkers and long-term eGFR in donors

Per SD increase in B2M, TIMP-2, IGFBP7, KIM-1 and NGAL 24 h after nephrectomy, donor eGFR at 3 months changed − 3.0 (− 6.5 to + 0.6), − 2.4 (− 4.7 to − 0.0), − 0.2 (− 2.7 to + 2.3), − 1.5 (− 4.0 to + 1.0) and − 3.1 (− 5.4 to − 0.8) ml/min/1.73m^2^, respectively. Per SD increase in B2M, TIMP-2, IGFBP7, KIM-1 and NGAL 24 h after nephrectomy, donor eGFR at 12 months changed − 1.1 (− 5.0 to + 2.6), − 2.3 (− 4.3 to + 0.1), − 0.7 (− 3.3 to + 1.9), − 1.6 (− 4.0 to + 0.9) and − 2.8 (− 5.1 to − 0.5) ml/min/1.73m^2^, respectively. Per SD increase in B2M, TIMP-2, IGFBP7, KIM-1 and NGAL 24 h after nephrectomy, the change in donor eGFR from 0 to 3 months was − 2.6 (− 8.6 to + 3.4), − 4.7 (− 8.6 to + 0.7), − 3.1 (− 7.2 to + 1.0), − 2.5 (− 6.7 to + 1.7) and − 3.1 (− 7.2 to + 1.1) ml/min/1.73m^2^ (Table 4). Results for these analyses with log-transformed biomarkers are shown in Supplemental Table 2.

**Table 4:** Linear regression analysis for relation between urinary biomarkers 24 h after nephrectomy and eGFR at 3 and 12 months in 38 (35 on + 12 months) living kidney donors.

	eGFR_cr_, mL/min/1.73 m^2^ (95% CI)		delta eGFR
	+ 3 months	+ 12 months	0 to + 3 months
B2M, µg/L, per 1 SD increase	− 3.0 (− 6.5 to + 0.6)	− 1.1 (− 5.0 to + 2.6)	− 2.6 (− 8.6 to + 3.4)
TIMP-2, pmol/L, per 1 SD increase	− 2.4 (− 4.7 to − 0.0)	− 2.3 (− 4.7 to + 0.1)	− 4.7 (− 8.6 to − 0.7)
IGFBP7, pmol/L, per 1 SD increase	− 0.2 (− 2.7 to + 2.3)	− 0.7 (− 3.3 to + 1.9)	− 3.1 (− 7.2 to + 1.0)
KIM-1, pg/mL, per 1 SD increase	− 1.5 (− 4.0 to + 1.0)	− 1.6 (− 4.0 to + 0.9)	− 2.5 (− 6.7 to + 1.7)
NGAL, pg/L, per 1 SD increase	− 3.1 (− 5.4 to − 0.8)	− 2.8 (− 5.1 to − 0.5)	− 3.1 (− 7.2 to + 1.1)

Results are presented as regression coefficients (95% CI).

*B2M* beta-2 microglobulin, *cr* creatinine, *IGFBP7* insulin-like growth factor-binding protein 7, *KIM-1* kidney Injury molecule-1, *NGAL* neutrophil gelatinase-associated lipocalin, *TIMP-2* tissue inhibitor of metalloproteinases-2.

### Association between biomarkers and long-term eGFR in recipients

Per SD increase in B2M, TIMP-2, IGFBP7, KIM-1 and NGAL 24 h after kidney transplantation, recipient eGFR at 3 months changed + 0.3 (− 4.4 to + 4.9), + 1.1 (− 4.3 to + 6.5), − 1.6 (− 7.0 to + 3.8), + 0.8 (− 4.7 to + 6.2) and − 2.4 (− 7.8 to + 2.9) ml/min/1.73m^2^, respectively. Per SD increase in B2M, TIMP-2, IGFBP7, KIM-1 and NGAL 24 h after kidney transplantation, recipient eGFR at 12 months changed − 1.3 (− 5.5 to + 2.8), + 0.4 (− 4.5 to + 5.3), − 0.5 (− 5.3 to + 4.4), − 0.6 (− 5.4 to + 4.3) and − 2.8 (− 7.6 to + 2.0) ml/min/1.73m^2^, respectively (Table 5). Results for these analyses with log-transformed biomarkers are shown in Supplemental Table 3.

**Table 5:** Linear regression analysis for relation between urinary biomarkers 24 h after kidney transplantation and eGFR at 3 and 12 months in 38 living recipients.

	eGFR_cr_, mL/min/1.73 m^2^ (95% CI)	
	+ 3 months	+ 12 months
B2M, µg/L, per SD increase	+ 0.3 (− 4.4 to + 4.9)	− 1.3 (− 5.5 to + 2.8)
TIMP-2, pmol/L, per SD increase	+ 1.1 (− 4.3 to + 6.5)	+ 0.4 (− 4.5 to + 5.3)
IGFBP7, pmol/L, per SD increase	− 1.6 (− 7.0 to + 3.8)	− 0.5 (− 5.3 to + 4.4)
KIM-1, pg/mL, per SD increase	+ 0.8 (− 4.7 to + 6.2)	− 0.6 (− 5.4 to + 4.3)
NGAL, pg/L, per SD increase	− 2.4 (− 7.8 to + 2.9)	− 2.8 (− 7.6 to + 2.0)

Results are presented as regression coefficients (95% CI).

*B2M* beta-2 microglobulin, *cr* creatinine, *IGFBP7* insulin-like growth factor-binding protein 7, *KIM-1* kidney Injury molecule-1, *NGAL* neutrophil gelatinase-associated lipocalin, *TIMP-2* tissue inhibitor of metalloproteinases-2.

## Discussion

In the 38 living donors investigated we found that, immediately after nephrectomy, urinary excretion of both B2M and NGAL levels increased significantly, similar to urinary albumin, while the excretion of TIMP-2 and IGFBP7 almost halved, similar to urinary creatinine. KIM-1 levels did not change considerably. None of these biomarkers showed a relation with short or long-term donor eGFR.

**Figure 2:**
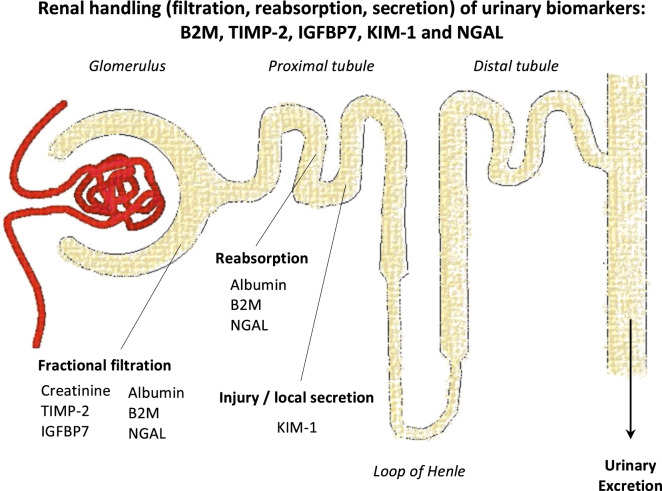
Cartoon depicting our hypothesis on urinary biomarker filtration, reabsorption and secretion based on the results of our measurements before and after living kidney transplantation in 38 living kidney donor–recipient pairs. Urinary creatinine, TIMP-2 and IGFBP7 levels halved immediately after nephrectomy in living kidney donors, suggesting these markers are predominantly indicative of glomerular filtration. Urinary albumin, B2M and NGAL increased significantly after nephrectomy, most likely mainly due to increased glomerular supply and decreased reabsorption by proximal tubular epithelial cells, e.g. due to megalin dysfunction after tubular injury. KIM1 only increased in recipients, possibly due to increased production following injury and subsequent secretion by proximal tubular cells following IRI. *B2M* beta-2 microglobulin, *IGFBP7* insulin-like growth factor-binding protein 7, *IRI* ischemiareperfusion injury, *KIM-1* kidney injury molecule-1, *NGAL* neutrophil gelatinase-associated lipocalin, *TIMP-2* tissue inhibitor of metalloproteinases-2.

Even though many studies have aimed to identify predictors of kidney injury, the clinical relevance and pathophysiological role of most biomarkers remains unclear^12^. To our knowledge, this is the first study that assessed TIMP-2, IGFBP7, KIM-1 and NGAL before and after unilateral nephrectomy in healthy donors. This population of otherwise healthy kidney donors provided the unique opportunity to explore the evolution of urinary biomarker immediately after a 50% reduction in nephron mass. For TIMP-2 and IGFBP7, we found that urinary levels almost halved after unilateral nephrectomy. The product of urinary TIMP-2 and IGFBP7, [TIMP-2] × [IGFBP7], has previously been identified as a predictor for stage 2 or 3 AKI, performing better than urinary NGAL, KIM-1, plasma Cystatin C or urinary TIMP-2 and IGFBP7 alone^11,18,19^. Follow-up studies could however not always validate these results^11,20^. TIMP-2 and IGFBP7 have previously been labelled as “cell cycle arrest biomarkers”, as they were known to induce G1 cell cycle arrest^11^. The relation between cell cycle arrest and TIMP-2 or IGBP7 has, however, never been specifically studied in kidney cells, and their physiological roles remain poorly understood^12^. Our finding that urinary TIMP-2 and IGFBP7 levels, similar to urinary creatinine levels, halved immediately after nephrectomy suggest that urinary excretion levels of TIMP-2 and IGFBP7 levels are primarily filtration dependent (Fig. 2). These results are in line with observations in experimental animals^21^. Furthermore, this may also explain why in previous research by our group, models containing only baseline plasma creatinine and duration of surgery performed equally well as compared to urinary [TIMP-2] x [IGFBP7] in predicting RRT after elective cardiac surgery^22^. Similarly, we previously showed that urinary levels of TIMP-2 as well as IGFBP7 were lower in patients with chronic kidney disease due to ADPKD as compared with healthy controls, even though beforehand it was hypothesized that urinary TIMP-2 levels should increase in patients with ADPKD in response to multiple repetitive acute ischemic events due to cyst growth and compression of surrounding tissue^23^.

In contrast, the excretion of urinary B2M and NGAL increased significantly after nephrectomy, similar to urinary albumin. In healthy kidneys, plasma B2M and NGAL are freely filtered by the glomeruli and nearly completely reabsorbed by the proximal tubular epithelial cells^12,24,25^. B2M is a molecule that associates with the major histocompatibility complex I (MHC-I)/human leukocyte antigen I (HLA-I) on the surface of all nucleated cells^24^. NGAL is a glycoprotein produced by epithelial tissues throughout the body, including distal tubular epithelial cells^24^. Both plasma B2M and NGAL can be expected to rise following any surgical procedure in case epithelial tissues are damaged. As B2M is more abundantly present in bodily tissues than NGAL, its increase would be expected to be larger. The rise in urinary B2M and NGAL excretion we observed, may reflect an increased supply of plasma B2M and NGAL to the glomeruli following the surgical procedure and/or decreased or insufficient reabsorption by proximal tubular epithelial cells immediately after halving the available nephron mass (Fig. 2). Of note, urinary albumin more than doubled the day after nephrectomy, which is too early to indicate compensatory hyperfiltration by the contralateral kidney^26,27^. Also, the change in urinary albumin in recipients compared to donors 24 h after transplantation was clearly larger than the change in B2M or urinary NGAL, B2M or fractional B2M excretion, potentially indicating urinary albumin to have a stronger relation with IRI. A direct comparison between donor and recipient urine levels of the biomarkers should be interpreted with caution, since except for B2M, both the blood concentration of the biomarkers in donor and recipient as well as the ultimate kidney function of the transplanted kidneys are unknown. Future studies with more frequent sampling may further explore the evolution of these biomarkers after nephrectomy in otherwise healthy donors and their relation to donor eGFR.

Urinary KIM-1 levels remained similar before and after nephrectomy in healthy kidney donors. KIM-1 is known to be a membrane glycoprotein produced by proximal tubular cells after ischaemic or nephrotoxic injury^11,25,28^. More importantly, it is not known to have a systemic source^11,25,28^. This may explain our finding that urinary KIM-1 levels did not change much after elective donor nephrectomy, but increased after additional reperfusion in recipients of transplanted kidneys as compared to their corresponding donors (Fig. 2).

We did not find a clear association between the urinary biomarkers we studied and short or long-term donor and recipient eGFR. In our donors, this finding may well be expected since in healthy donors the remaining kidneys are not supposed to be significantly damaged by ischemia during nephrectomy of the contralateral kidney. Obviously, kidney injury may occur in some donors due to unintended hypoperfusion during surgery or use of nephrotoxic drugs^29^. However, in our small study sample, we did not expect to be able to measure this potential effect. In contrast, the kidneys transplanted to our recipients experienced additional reperfusion injury during the transplant procedure. However, a single measure of potential kidney injury biomarkers on the first day after transplantation will not likely reflect longer-term recipient eGFR, since renal function is obviously highly affected by other additional factors, such as the occurrence of acute rejection episodes and/or consequences of immunosuppressant drugs and their inherent potential nephrotoxic effects.

There are several strengths to our study. First, this is the first study investigating the levels of urinary B2M, TIMP-2, IGFBP7, NGAL and KIM-1 excretion before and after nephrectomy in otherwise healthy donors. These results can therefore provide valuable insights in the evolution of these urinary biomarkers in the general population. Second, by including the kidney recipients corresponding to these donors, we were also able to directly assess these biomarker levels after reperfusion injury in the same kidneys after transplantation. Third, we calculated the short-term eGFR using both plasma creatinine and cystatin C, allowing for a more precise estimation^17^. This enabled us to better interpret the results of our urinary biomarker measurements.

Nevertheless, our study has some limitations. First, due to the limited sample size, this study may have been underpowered, particularly for detecting associations between urinary levels of B2M, TIMP-2, IGFBP7, NGAL and KIM-1 and concurrent kidney function and kidney function decline. This will however not have inferred with our descriptive analysis assessing the patterns of urinary B2M, TIMP-2, IGFBP7, NGAL and KIM-1 evolution before and after nephrectomy in otherwise healthy donors. Second, all urine samples included in our analysis were stored for 7–10 years. Even though they only underwent one freeze–thaw cycle, this may have affected the sensitivity of our additional measurements to some extent. Third, as stated above, a direct comparison between donor and recipient urine levels of the biomarkers should be interpreted with caution, since except for B2M, both the blood concentration of the biomarkers in donor and recipient as well as the ultimate kidney function of the transplanted kidneys are unknown. Furthermore, as opposed to healthy donors, recipients have end-stage kidney disease before kidney transplantation, may suffer immunologic and/or hemodynamic injury, and are treated with immunosuppressants.

In conclusion, we found that urinary TIMP-2 and IGFBP7 excretion halved immediately after nephrectomy in living kidney donors, similar to the urinary pattern of creatinine excretion, suggesting these markers are predominantly indicative of glomerular filtration with or without some tubular secretion. In contrast, the excretion of urinary B2M and NGAL increased significantly after nephrectomy, similar to urinary albumin, most likely mainly due to decreased reabsorption by proximal tubular epithelial cells. KIM-1 did not change considerably after unilateral nephrectomy in healthy kidney donors. None of these biomarkers showed a strong relation with short or long-term donor eGFR. Similar biomarker patterns were seen in the recipients of these transplanted kidneys, except for KIM-1, which was increased, most likely following the additional reperfusion injury.

### Supplementary Information

Supplementary Tables.

## Data Availability

As our data could be used to identify individuals, privacy concerns prevent us from allowing them to be publically available. Nonetheless, we are open to make our data available for collaboration conditional on agreement on privacy matters and appropriate usage of the data. For this, please contact the corresponding author.
