# Supplementary material for: Urinary beta-2 microglobulin increases whereas TIMP-2 and IGFBP7 decline after unilateral nephrectomy in healthy kidney donors
Source: Sci Rep. 2024 Jun 5;14:12901. doi: 10.1038/s41598-024-62246-1 (PMC11153551; doi:10.1038/s41598-024-62246-1)
Supplement: Supplementary file 1 — Supplementary Tables. [file 41598_2024_62246_MOESM1_ESM.docx]

| **Supplemental Table 1.** Linear regression analysis for association between log-transformed urinary creatinine standardized urinary TIMP-2, IGFBP7, KIM-1 or NGAL, and CKD-EPI eGFR based on serum creatinine and cystatin C 24 hours before and after living kidney transplantation in 38 donor-recipient pairs, adjusted for donor age and sex. | | | |
| --- | --- | --- | --- |
|  | **Donor age and sex adjusted** | | |
|  | **eGFR_cr-cysC_, mL/min/1.73 m^2^ (95% CI)** | | |
|  | Donor | | Recipient |
|  | 24h prior | 24h post | 24h post |
|  | *n* = 37* | *n* = 38 | *n* = 37* |
| Log(B2M/Creat) | -8.0 (-12.1 to -3.9) | +0.9 (-1.1 to +2.9) | -0.1 (-3.5 to +3.4) |
|  |  |  |  |
| Log(TIMP-2/Creat) | +4.5 (-4.0 to +13.0) | +4.8 (-6.5 to +16.1) | +10.0 (-1.2 to +21.1) |
|  |  |  |  |
| Log(IGFBP7/Creat) | +8.0 (+0.3 to +15.7) | +2.3 (-5.4 to +9.9) | +12.9 (2.4 to +23.3) |
|  |  |  |  |
| Log(KIM-1/Creat) | -3.2 (-8.7 to +2.3) | -1.6 (-5.3 to +2.1) | +7.8 (3.2 to +12.5) |
|  |  |  |  |
| Log(NGAL/Creat) | +0.5 (-4.4 to +5.4) | -2.4 (-4.9 to +0.1) | +3.3 (-1.2 to +7.8) |
|  |  |  |  |
| Log(ACR, mg/mmol) | -0.8 (-20.5 to +18.9) | +2.9 (-1.8 to 7.7) | +1.8 (-4.4 to 8.0) |
| Results are presented as linear regression coefficients (95% CI). ACR: albumin-to-creatinine ratio, cr: creatinine, cysC: cystatin C, IGFBP7: insulin-like growth factor-binding protein 7, KIM-1: kidney Injury molecule-1, NGAL: Neutrophil gelatinase-associated lipocalin, TIMP-2: tissue inhibitor of metalloproteinases-2. *Serum samples were missing for 1 donor at 24h before and 1 recipient at 24h after living kidney transplantation. | | | |

**Supplemental Material**

| **Supplemental Table 2.** Linear regression analysis for relation between log-transformed urinary biomarkers 24 hours after nephrectomy and eGFR at 3 and 12 months in 38 (35 on +12 months) living kidney donors. | | | |
| --- | --- | --- | --- |
|  | **eGFR_cr_, mL/min/1.73 m^2^ (95% CI)** | | **delta eGFR** |
|  | **+3 months** | **+12 months** | **0 to +3 months** |
| Log(B2M, µg/L), per 1 unit increase | -0.7 (-2.4 to + 1.1) | -0.1 (-1.9 to +1.7) | -0.1 (-3.0 to +2.8) |
|  |  |  |  |
| Log(TIMP2, pmol/L), per 1 unit increase | -2.5 (-5.4 to + 0.3) | -2.4 (-5.3 to +0.5) | -6.7 (-11.3 to -2.2) |
|  |  |  |  |
| Log(IGFBP7, pmol/L), per 1 unit increase | -1.8 (-6.7 to +3.1) | -2.2 (-7.3 to + 2.9) | -8.0 (-16.0 to +0.1) |
|  |  |  |  |
| Log(KIM-1, pg/mL), per 1 unit increase | -1.5 (-3.7 to + 0.6) | -1.6 (-3.7 to + 0.5) | -3.8 (-7.3 to -0.3) |
|  |  |  |  |
| Log(NGAL, pg/L), per 1 unit increase | -1.9 (-3.5 to -0.2) | -1.4 (-3.1 to + 0.3) | -2.5 (-5.3 to +0.4) |
| Results are presented as regression coefficients (95% CI). B2M: beta-2 microglobulin, cr: creatinine, IGFBP7: insulin-like growth factor-binding protein 7, KIM-1: kidney Injury molecule-1, NGAL: Neutrophil gelatinase-associated lipocalin, TIMP-2: tissue inhibitor of metalloproteinases-2. | | | |

| **Supplemental Table 3.** Linear regression analysis for relation between log-transformed urinary biomarkers 24 hours after kidney transplantation and eGFR at 3 and 12 months in 38 living recipients. | | |
| --- | --- | --- |
|  | **eGFR_cr_, mL/min/1.73 m^2^ (95% CI)** | |
|  | **+3 months** | **+12 months** |
| Log(B2M, µg/L), per unit increase | -0.4 (-4.6 to +3.8) | -2.0 (-5.8 to +1.7) |
|  |  |  |
| Log(TIMP2, pmol/L), per unit increase | -0.0 (-8.7 to +8.6) | +0.1 (-7.8 to +7.9) |
|  |  |  |
| Log(IGFBP7, pmol/L), per unit increase | -3.6 (-17.7 to +10.5) | -1.5 (-14.2 to +11.3) |
|  |  |  |
| Log(KIM-1, pg/mL), per unit increase | +1.0 (-5.1 to +7.1) | -1.8 (-7.3 to +3.7) |
|  |  |  |
| Log(NGAL, pg/L), per unit increase | -2.0 (-6.0 to +2.0) | -2.6 (-6.1 to +1.0) |
| Results are presented as regression coefficients (95% CI). B2M: beta-2 microglobulin, cr: creatinine, IGFBP7: insulin-like growth factor-binding protein 7, KIM-1: kidney Injury molecule-1, NGAL: Neutrophil gelatinase-associated lipocalin, TIMP-2: tissue inhibitor of metalloproteinases-2. | | |
